# 1-(5-Chloro-6-fluoro-1,3-benzothia­zol-2-yl)hydrazine

**DOI:** 10.1107/S160053681203156X

**Published:** 2012-07-18

**Authors:** Hoong-Kun Fun, Ching Kheng Quah, B. K. Sarojini, B. J. Mohan, B. Narayana

**Affiliations:** a X-ray Crystallography Unit, School of Physics, Universiti Sains Malaysia, 11800 USM, Penang, Malaysia; b Department of Chemistry, P. A. College of Engineering, Nadupadavu, Mangalore 574 153, India; c Department of Chemistry, Mangalore University, Mangalagangotri 574 199, Mangalore, India

## Abstract

In the title compound, C_7_H_5_ClFN_3_S, the 1,3-benzothia­zole ring system is nearly planar (r.m.s. deviation = 0.023 Å). In the crystal, mol­ecules are linked *via* inter­molecular N—H⋯N hydrogen bonds into a two-dimensional network parallel to (100).

## Related literature

For general background to and the biological activities of benzothia­zole derivatives, see: Yaseen *et al.* (2006 ▶); Kini *et al.* (2007 ▶); Munirajasekhar *et al.* (2011 ▶); Gurupadayya *et al.* (2008 ▶); Bowyer *et al.* (2007 ▶); Mittal *et al.* (2007 ▶); Pozas *et al.* (2005 ▶); Rana *et al.* (2008 ▶). For a related structure, see: Fun *et al.* (2012 ▶). For standard bond-length data, see: Allen *et al.* (1987 ▶). For the stability of the temperature controller used for the data collection, see: Cosier & Glazer (1986 ▶).
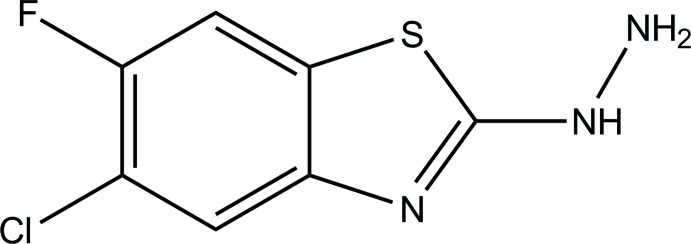


## Experimental

### 

#### Crystal data

C_7_H_5_ClFN_3_S
*M* *_r_* = 217.65
Monoclinic, 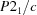

*a* = 11.1287 (6) Å
*b* = 5.6641 (3) Å
*c* = 13.3419 (7) Å
β = 108.552 (1)°
*V* = 797.29 (7) Å^3^
*Z* = 4
Mo *K*α radiation
μ = 0.70 mm^−1^
*T* = 100 K
0.31 × 0.16 × 0.14 mm

#### Data collection

Bruker SMART APEXII DUO CCD area-detector diffractometer
Absorption correction: multi-scan (*SADABS*; Bruker, 2009 ▶) *T*_min_ = 0.813, *T*_max_ = 0.908
9459 measured reflections
2899 independent reflections
2638 reflections with *I* > 2σ(*I*)
*R*_int_ = 0.017

#### Refinement

*R*[*F* ^2^ > 2σ(*F* ^2^)] = 0.024
*wR*(*F* ^2^) = 0.066
*S* = 1.07
2899 reflections
138 parameters
All H-atom parameters refined
Δρ_max_ = 0.53 e Å^−3^
Δρ_min_ = −0.20 e Å^−3^

### 

Data collection: *APEX2* (Bruker, 2009 ▶); cell refinement: *SAINT* (Bruker, 2009 ▶); data reduction: *SAINT*; program(s) used to solve structure: *SHELXTL* (Sheldrick, 2008 ▶); program(s) used to refine structure: *SHELXTL*; molecular graphics: *SHELXTL*; software used to prepare material for publication: *SHELXTL* and *PLATON* (Spek, 2009 ▶).

## Supplementary Material

Crystal structure: contains datablock(s) global, I. DOI: 10.1107/S160053681203156X/sj5255sup1.cif

Structure factors: contains datablock(s) I. DOI: 10.1107/S160053681203156X/sj5255Isup2.hkl

Supplementary material file. DOI: 10.1107/S160053681203156X/sj5255Isup3.cml

Additional supplementary materials:  crystallographic information; 3D view; checkCIF report

## Figures and Tables

**Table 1:** Hydrogen-bond geometry (Å, °)

*D*—H⋯*A*	*D*—H	H⋯*A*	*D*⋯*A*	*D*—H⋯*A*
N2—H1N2⋯N1^i^	0.816 (16)	2.132 (16)	2.9478 (12)	176.9 (16)
N3—H2N3⋯N3^ii^	0.850 (16)	2.443 (17)	3.1382 (12)	139.5 (14)

Symmetry codes: (i) 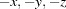; (ii) 
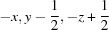
.

**Fig. 1.**
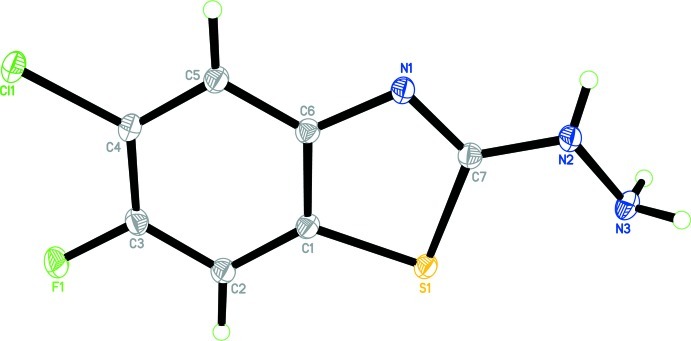
The molecular structure of the title compound showing 50% probability displacement ellipsoids for non-H atoms.

**Fig. 2.**
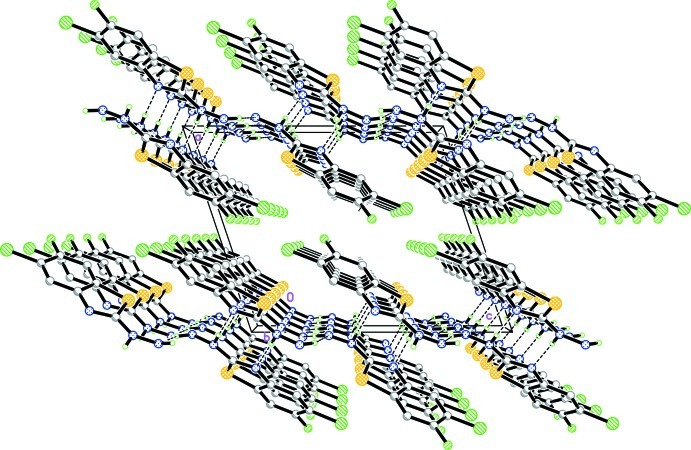
The crystal structure of the title compound, viewed along the b axis. H atoms not involved in hydrogen bonds (dashed lines) have been omitted for clarity.

## supplementary crystallographic information

### Comment

Benzothiazoles are very important bicyclic ring compounds which are of great interest because of their biological activities. The substituted benzothiazole derivatives have emerged as significant components in various diversified therapeutic applications. A literature review reveals that benzothiazoles and their derivatives show considerable activity, including potent inhibition of human immunodeficiency virus type 1 (HIV-1) replication by HIV-1 protease inhibition (Yaseen *et al.*, 2006), antitumor (Kini *et al.*, 2007), anthelmintic (Munirajasekhar *et al.*, 2011), analgesic and anti-inflammatory (Gurupadayya *et al.*, 2008), antimalarial (Bowyer *et al.*, 2007), antifungal (Mittal *et al.*, 2007), anticandidal activities (Pozas *et al.*, 2005) and various activities relating to the central nervous system (Rana *et al.*, 2008).

In the title molecule (Fig. 1), the benzo[d]thiazol-2-yl ring system (S1/N1/C1–C7) is nearly planar (r.m.s. deviation = 0.023). Bond lengths (Allen *et al.*, 1987) and angles are within normal ranges and are comparable with a related structure (Fun *et al.*, 2012).

In the crystal structure, Fig. 2, molecules are linked *via* intermolecular N2—H1N2···N1 and N3—H2N3···N3 hydrogen bonds (Table 1) into two-dimensional networks parallel to (100).

### Experimental

Concentrated HCl (6 ml) was added drop-wise to hydrazine hydrate [6 ml, 0.12 mol] at 273–283 K followed by ethylene glycol (50 ml). To the above solution, 5-chloro-6-fluoro benzothiazol-2-amine [6.079 g, 0.03 mol] was added in portions. It was then refluxed for 3–4 h. A colourless solid was precipitated at the end of the reflux period. The mixture was cooled and the product was filtered and then washed with water several times. It was air dried and recrystallized using ethanol. The single crystals were grown by slow evaporation from solvent methanol (m.p. = 483–485 K).

### Refinement

All hydrogen atoms were located in a difference Fourier map and refined freely with N—H = 0.815 (16)–0.905 (15) Å and C—H = 0.951 (14) or 0.966 (15) Å.

### Crystal data

**Table:**

C_7_H_5_ClFN_3_S	*F*(000) = 440
*M**_r_* = 217.65	*D*_x_ = 1.813 Mg m^−^^3^
Monoclinic, *P*2_1_/*c*	Mo *K*α radiation, λ = 0.71073 Å
Hall symbol: -P 2ybc	Cell parameters from 5638 reflections
*a* = 11.1287 (6) Å	θ = 3.9–32.6°
*b* = 5.6641 (3) Å	µ = 0.70 mm^−^^1^
*c* = 13.3419 (7) Å	*T* = 100 K
β = 108.552 (1)°	Block, colourless
*V* = 797.29 (7) Å^3^	0.31 × 0.16 × 0.14 mm
*Z* = 4	

### Data collection

**Table:**

Bruker SMART APEXII DUO CCD area-detector diffractometer	2899 independent reflections
Radiation source: fine-focus sealed tube	2638 reflections with *I* > 2σ(*I*)
Graphite monochromator	*R*_int_ = 0.017
φ and ω scans	θ_max_ = 32.7°, θ_min_ = 1.9°
Absorption correction: multi-scan (*SADABS*; Bruker, 2009)	*h* = −16→16
*T*_min_ = 0.813, *T*_max_ = 0.908	*k* = −8→8
9459 measured reflections	*l* = −20→20

### Refinement

**Table:**

Refinement on *F*^2^	Primary atom site location: structure-invariant direct methods
Least-squares matrix: full	Secondary atom site location: difference Fourier map
*R*[*F*^2^ > 2σ(*F*^2^)] = 0.024	Hydrogen site location: inferred from neighbouring sites
*wR*(*F*^2^) = 0.066	All H-atom parameters refined
*S* = 1.07	*w* = 1/[σ^2^(*F*_o_^2^) + (0.0319*P*)^2^ + 0.296*P*] where *P* = (*F*_o_^2^ + 2*F*_c_^2^)/3
2899 reflections	(Δ/σ)_max_ = 0.001
138 parameters	Δρ_max_ = 0.53 e Å^−^^3^
0 restraints	Δρ_min_ = −0.20 e Å^−^^3^

### Special details

**Table:**

Experimental. The crystal was placed in the cold stream of an Oxford Cryosystems Cobra open-flow nitrogen cryostat (Cosier & Glazer, 1986) operating at 100.0 (1) K.
Geometry. All esds (except the esd in the dihedral angle between two l.s. planes) are estimated using the full covariance matrix. The cell esds are taken into account individually in the estimation of esds in distances, angles and torsion angles; correlations between esds in cell parameters are only used when they are defined by crystal symmetry. An approximate (isotropic) treatment of cell esds is used for estimating esds involving l.s. planes.
Refinement. Refinement of F^2^ against ALL reflections. The weighted R-factor wR and goodness of fit S are based on F^2^, conventional R-factors R are based on F, with F set to zero for negative F^2^. The threshold expression of F^2^ > 2sigma(F^2^) is used only for calculating R-factors(gt) etc. and is not relevant to the choice of reflections for refinement. R-factors based on F^2^ are statistically about twice as large as those based on F, and R-factors based on ALL data will be even larger.

### Fractional atomic coordinates and isotropic or equivalent isotropic displacement parameters (Å^2^)

**Table:**

	*x*	*y*	*z*	*U*_iso_*/*U*_eq_	
S1	0.17661 (2)	0.55946 (4)	0.127235 (17)	0.01252 (6)	
F1	0.45495 (6)	0.83076 (11)	−0.09724 (5)	0.01932 (13)	
Cl1	0.40693 (2)	0.42019 (4)	−0.236217 (18)	0.01702 (6)	
N1	0.13780 (7)	0.19036 (15)	0.00140 (6)	0.01316 (14)	
N2	0.02947 (8)	0.17861 (16)	0.12535 (6)	0.01531 (15)	
N3	−0.01178 (8)	0.31566 (15)	0.19707 (6)	0.01435 (15)	
C1	0.25396 (8)	0.54873 (16)	0.03242 (7)	0.01196 (15)	
C2	0.33315 (8)	0.71802 (17)	0.01037 (7)	0.01342 (15)	
C3	0.37807 (8)	0.67098 (17)	−0.07310 (7)	0.01365 (16)	
C4	0.34704 (8)	0.46473 (17)	−0.13335 (7)	0.01302 (15)	
C5	0.26897 (8)	0.29589 (17)	−0.11025 (7)	0.01283 (15)	
C6	0.22136 (8)	0.33884 (16)	−0.02676 (7)	0.01158 (15)	
C7	0.10783 (8)	0.28418 (17)	0.08008 (7)	0.01228 (15)	
H2A	0.3595 (12)	0.862 (3)	0.0500 (11)	0.014 (3)*	
H5A	0.2461 (12)	0.158 (3)	−0.1530 (11)	0.015 (3)*	
H1N2	−0.0159 (14)	0.073 (3)	0.0922 (12)	0.023 (4)*	
H1N3	−0.0937 (14)	0.360 (3)	0.1679 (12)	0.023 (4)*	
H2N3	−0.0068 (14)	0.231 (3)	0.2508 (12)	0.025 (4)*	

### Atomic displacement parameters (Å^2^)

**Table:**

	*U*^11^	*U*^22^	*U*^33^	*U*^12^	*U*^13^	*U*^23^
S1	0.01537 (10)	0.01201 (11)	0.01173 (10)	−0.00092 (7)	0.00648 (7)	−0.00130 (7)
F1	0.0220 (3)	0.0177 (3)	0.0227 (3)	−0.0073 (2)	0.0133 (2)	−0.0019 (2)
Cl1	0.01930 (11)	0.01979 (12)	0.01571 (10)	−0.00142 (8)	0.01083 (8)	−0.00148 (8)
N1	0.0149 (3)	0.0130 (3)	0.0136 (3)	−0.0015 (3)	0.0075 (3)	−0.0013 (3)
N2	0.0196 (4)	0.0146 (4)	0.0155 (3)	−0.0048 (3)	0.0108 (3)	−0.0036 (3)
N3	0.0165 (3)	0.0160 (4)	0.0128 (3)	0.0018 (3)	0.0079 (3)	0.0001 (3)
C1	0.0131 (3)	0.0121 (4)	0.0113 (3)	0.0002 (3)	0.0048 (3)	−0.0004 (3)
C2	0.0149 (4)	0.0121 (4)	0.0142 (4)	−0.0013 (3)	0.0060 (3)	−0.0008 (3)
C3	0.0133 (3)	0.0136 (4)	0.0153 (4)	−0.0018 (3)	0.0062 (3)	0.0007 (3)
C4	0.0134 (3)	0.0150 (4)	0.0122 (3)	0.0010 (3)	0.0063 (3)	0.0002 (3)
C5	0.0134 (3)	0.0136 (4)	0.0124 (3)	0.0004 (3)	0.0054 (3)	−0.0007 (3)
C6	0.0124 (3)	0.0112 (4)	0.0118 (3)	0.0003 (3)	0.0047 (3)	−0.0002 (3)
C7	0.0133 (3)	0.0120 (4)	0.0121 (3)	−0.0004 (3)	0.0050 (3)	0.0003 (3)

### Geometric parameters (Å, º)

**Table:**

S1—C1	1.7429 (9)	N3—H2N3	0.849 (17)
S1—C7	1.7625 (10)	C1—C2	1.3957 (13)
F1—C3	1.3529 (11)	C1—C6	1.4093 (13)
Cl1—C4	1.7243 (9)	C2—C3	1.3839 (12)
N1—C7	1.3109 (11)	C2—H2A	0.966 (15)
N1—C6	1.3912 (11)	C3—C4	1.3977 (13)
N2—C7	1.3483 (11)	C4—C5	1.3910 (13)
N2—N3	1.4172 (11)	C5—C6	1.3986 (12)
N2—H1N2	0.815 (16)	C5—H5A	0.951 (14)
N3—H1N3	0.905 (15)		
			
C1—S1—C7	88.21 (4)	F1—C3—C4	118.78 (8)
C7—N1—C6	109.48 (8)	C2—C3—C4	122.51 (9)
C7—N2—N3	117.01 (8)	C5—C4—C3	120.30 (8)
C7—N2—H1N2	117.3 (11)	C5—C4—Cl1	120.18 (7)
N3—N2—H1N2	119.3 (11)	C3—C4—Cl1	119.52 (7)
N2—N3—H1N3	111.1 (10)	C4—C5—C6	118.59 (8)
N2—N3—H2N3	108.3 (11)	C4—C5—H5A	119.8 (8)
H1N3—N3—H2N3	107.7 (14)	C6—C5—H5A	121.5 (8)
C2—C1—C6	121.92 (8)	N1—C6—C5	124.41 (8)
C2—C1—S1	128.31 (7)	N1—C6—C1	115.70 (8)
C6—C1—S1	109.73 (7)	C5—C6—C1	119.85 (8)
C3—C2—C1	116.83 (9)	N1—C7—N2	122.99 (9)
C3—C2—H2A	118.6 (8)	N1—C7—S1	116.89 (7)
C1—C2—H2A	124.6 (8)	N2—C7—S1	120.11 (7)
F1—C3—C2	118.72 (8)		
			
C7—S1—C1—C2	177.55 (9)	C7—N1—C6—C1	−0.18 (11)
C7—S1—C1—C6	0.05 (7)	C4—C5—C6—N1	176.84 (8)
C6—C1—C2—C3	0.32 (13)	C4—C5—C6—C1	−0.63 (13)
S1—C1—C2—C3	−176.91 (7)	C2—C1—C6—N1	−177.63 (8)
C1—C2—C3—F1	179.88 (8)	S1—C1—C6—N1	0.06 (10)
C1—C2—C3—C4	−0.13 (14)	C2—C1—C6—C5	0.06 (14)
F1—C3—C4—C5	179.54 (8)	S1—C1—C6—C5	177.75 (7)
C2—C3—C4—C5	−0.45 (14)	C6—N1—C7—N2	−179.09 (8)
F1—C3—C4—Cl1	−0.12 (12)	C6—N1—C7—S1	0.22 (10)
C2—C3—C4—Cl1	179.89 (7)	N3—N2—C7—N1	−169.63 (8)
C3—C4—C5—C6	0.82 (13)	N3—N2—C7—S1	11.09 (11)
Cl1—C4—C5—C6	−179.52 (7)	C1—S1—C7—N1	−0.16 (8)
C7—N1—C6—C5	−177.75 (8)	C1—S1—C7—N2	179.17 (8)

### Hydrogen-bond geometry (Å, º)

**Table:**

*D*—H···*A*	*D*—H	H···*A*	*D*···*A*	*D*—H···*A*
N2—H1*N*2···N1^i^	0.816 (16)	2.132 (16)	2.9478 (12)	176.9 (16)
N3—H2*N*3···N3^ii^	0.850 (16)	2.443 (17)	3.1382 (12)	139.5 (14)

Symmetry codes: (i) −*x*, −*y*, −*z*; (ii) −*x*, *y*−1/2, −*z*+1/2.
